# Effect of Epley's maneuver on the quality of life of paroxismal positional benign vertigo patients

**DOI:** 10.1590/S1808-86942010000600006

**Published:** 2015-10-19

**Authors:** Alcione Botelho Pereira, Juliana Nunes Santos, Fernando Madalena Volpe

**Affiliations:** 1 Speech therapist; 2 Doctoral degree in Health Sciences, full professor of speech therapy, FEAD; 3 Master's degree in epidemiology, doctoral degree in psychiatry, coordinator of research projects in health, FHEMIG. FEAD - Entrepreneurial Management Center (Centro de Gestão Empreendedora) and FHEMIG - Hospital Foundation for the State of Minas Gerais (Fundação Hospitalar do Estado de Minas Gerais)

**Keywords:** vestibular diseases, quality of life, rehabilitation, vertigo

## Abstract

Quality of life (QoL) is significantly impaired by vertigo. The effect of specific treatments on QoL deserves investigation.

**Aim:**

To assess the effect of repositioning maneuvers on the QoL of benign paroxysmal positioning vertigo (BPPV) patients.

**Materials and Methods:**

A retrospective study design consiting of reviews of charts of BPPV patients in a vestibular rehabilitation unit at a teaching institution in Belo Horizonte, MG, Brazil, from 2007 to 2008. Pre- and post-therapy (Epley's repositioning maneuver) scores on the physical, functional and emotional dimensions of the Dizziness Handicap Inventory (DHI) were analyzed.

**Results:**

Twenty-one patients were included, eighteen (86%) were females; the average age was 53.2 years. Ten patients presented bilateral BPPV; in eleven it was unilateral. The mean interval between assessments (pre- and post-treatment) was 21 days. The average number of required maneuvers was 2.3 (±1.1). Pre-treatment DHI results showed a significant impact of BPPV on quality of life. Initial scores for physical (17.5), functional (17.3), emotional (13.2) dimensions decreased with therapy: respectively 3.7, 3.9, and 3.2 (p<0.001).

**Conclusion:**

In the present sample, Epley's maneuver had a positive and significant effect on emotional, physical and functional dimensions of quality of life, as measured by the DHI scores before and after therapy.

## INTRODUCTION

Benign paroxysmal positional vertigo (BPPV) is one of the most common disorders of the vestibular system, present in 19% of patients with dizziness;^1^, ^2^ it may be unilateral or involve both labyrinths.^3^ BPPV may be characterized by the symptom rotating dizziness caused by sudden changes in the position of the head, such as lying down on one or both sides, moving into an orthostatic position or looking upwards.^4^ Other accompanying symptoms may be nausea, vomiting and positioning nystagmus,^1^ which occur unpredictably and suddenly.^5^

Several theories have attempted to explain the pathophysiology of BPPV. These theories may be divided essentially into two currents: cupulolithiasis and ductolithiasis.^6^ In cupulolithiasis, degenerated otoconial fragments in the utricle adhere to the cupula of the posterior semicircular canal, making it more dense than the surrounding endolymph, and thus more susceptible to the effects of gravity.^7^ The ductal lithiasis or canalithiasis theory contends that degenerated fragments do not adhere to the cupula, but remain floating in the endolymph of the posterior canal.^8^ In both theories, head movements cause the fragments to move, which stimulates the cupula of the posterior semicircular canal inappropriately and excites the posterior ampullary nerve, resulting in vertigo.^7^

The etiology of BPPV may involve cranial trauma, metabolic diseases, hormone dysfunction, and other conditions;^4^, ^8^ in most cases, however, BPPV is idiopathic.^9^

There are several approaches to treat BPPV, such as vestibular habituation exercises, labyrinth sedation drugs, surgical ablation of the posterior semicircular canal, and repositioning maneuvers, which has been more widely adopted than the former methods.^10^ The most commonly used approach for the treatment of BPPV is Epley's maneuver; it results in rapid short-term improvements.^11^

The diagnosis and treatment of BPPV can significantly improve the quality of life (QoL) of patients.^12^

Vestibular conditions may have a negative impact on the daily activities of patients. Thus studies to assess the main emotional and/or social aspects are required for more appropriate rehabilitation according to the individual needs of patients.^3^

The purpose of this study was to investigate the effect of Epley's maneuver on the QoL of BPPV patients seen at a speech therapy clinic in Belo Horizonte, Minas Gerais, Brazil.

## MATERIAL AND METHOD

The institutional review board of the institution approved this study on 22 February 2008.

An observational retrospective cohort study was carried out. The sample consisted of the charts of BPPV patients treated in the vestibular rehabilitation unit of the speech therapy clinic from February 2007 to November 2008.

The exclusion criteria were not answering the dizziness handicap inventory (DHI) and/or not signing the free informed consent form.

Before therapy patients routinely receive explanations about the school-clinic, its teaching, clinical research and outreach programs and sign a free informed consent form. All patients undergoing vestibular rehabilitation routinely answer the Portuguese translated DHI before and after the treatment. The DHI is a specific questionnaire for assessing the impact of dizziness on the QoL of patients; it assesses the self-perception of the incapacitating effects of dizziness.^13^ The DHI consists of 25 questions; 7 questions deal with physical aspects, 9 questions evaluate the emotional aspects, and a further 9 questions assess the functional aspects. Patients answer “yes”, “no” or “sometimes”. “Yes” answers score four points, “no” answers score zero, and “sometimes” scores two points. The maximum score is 28 for the physical aspects, 36 points for the emotional aspects, and 36 points for the functional aspects, totaling 100 points. Higher scores are associated with more losses in the QoL of subjects; the questions investigate the self-perception of patients about the difficulties caused by dizziness on their daily activities.^3^, ^13^, ^14^

In BPPV cases, the Dix-Hallpike test is conducted for diagnosis, after which Epley's maneuver is applied for repositioning the otoconia. These procedures are described below:

The Dix-Hallpike positional test consists of moving the patient's head to cause movement of the endolymph, thereby moving the cupula of the posterior semicircular canal. In this maneuver, patients are seated with the head rotated laterally about 45 degrees (right or left, depending on which side is to be tested). The examiner holds the patient's head and rapidly moves the patient into dorsal decubitus with the head tilted backwards about 30 degrees. The patient stays in this position with eyes open and a fixed gaze. In BPPV patients, a short latency period is followed by nystagmus^15^ with positioning vertigo and neurovegetative signs.^1^, ^4^, ^5^, ^8^

Epley's maneuver consists of seating the patient on an examining bed in such as way that upon decubitus the head will be above the end of the bed. The examiner then moves the patient into dorsal decubitus with the head extended and rotated 45 degrees laterally towards the affected labyrinth (where the Dix-Hallpike test is positive), after which the head is rotated 90 degrees to the opposite side; at this point, the patient is helped into a lateral decubitus position gazing towards the floor. The patient is then returned to a sitting position with the head rotated, after which the head is returned to its initial position, gazing forward and with the head tilted forward 20 degrees.^16^

A routine procedure for analyzing the data in charts included the variables age, sex, duration of treatment, number of maneuvers done, uni- or bidirectional maneuvers, physical/functional/emotional scores (before and after therapy), and total DHI score.

The Minitab software version 13.01 was used to build a specific data base for this study. The descriptive analysis consisted of studying the distribution of categorical variables, and the central tendency measures (mean, median and the standard deviation) of continuous variables. Inferential analysis consisted of Student's t test for paired data (p < 0.05).

## RESULTS

Two of 23 patient charts did not contain DHI results after repositioning therapy with Epley's maneuver.

The study sample comprised 21 patients, of which 18 were female (86%). The mean age of patients was 53.2 years (± 20.0). The diagnostic test for BPPV was positive on one side only in 11 patients; it was bilateral in the other 10 patients. The median time interval between DHI questionnaires applied before and after therapy was 21 days. Among 18 patients, 6 were treated with one maneuver, 7 were treated with two maneuvers, 5 were treated with three maneuvers, 2 were treated with four maneuvers, and 1 patient was treated with five maneuvers; the mean number of maneuvers was 2.3 (± 1.2).

Among the DHI items in 21 patients, the physical findings were the most compromised aspects, followed by the functional and the emotional aspects.

Table 1 shows the total DHI scores before and after therapy.

**Table 1:** Results of the vertigo inventory in 21 BPPV patients before and after therapy.

	Before		After		Difference	t	p*
Results	Mean	Standard deviation	Mean	Standard deviation			
Physical score	17,48	6,00	3,71	5,49	13,76	8,55	<0,001
Functional score	17,29	9,61	3,95	6,15	13,33	6,01	<0,001
Emotional score	13,19	8,81	3,24	7,14	9,95	5,40	<0,001
Total score	48,05	19,47	10,90	16,33	37,14	8,50	<0,001

* Paired t test

## DISCUSSION

The Portuguese translated and validated DHI was chosen to study the harmful effects of dizziness in the study population. It is a focused questionnaire that has been translated and adapted for use in the Brazilian population;^13^ it is also easy to understand and to apply.

It is worth noting that the sample was relatively small (n=21) compared to those in other studies on the effect of dizziness on the QoL of BPPV patients (n=42^17^ and n=70^14^); there was also no control group for comparison purposes. The results, therefore, should not be considered as definitive, but rather as suggesting a relation between BPPV and QoL.

There were more altered results in females compared to males in this study. Published papers have suggested that altered hormone levels - more frequent in women - may raise the occurrence of BPPV.^1^, ^4^ Other studies of vestibular disorders have also noted a predominance of female patients, ranging from 70 to 89%.^8^, ^14^, ^18^, ^19^ These findings corroborate our results. The age ranged from 21 to 82 years, similar to that in other published papers.^2^, ^7^, ^20^, ^21^

The mean number of maneuvers needed to abolish positioning nystagmus was 2.3. This finding is similar to that in another study in which the mean number of required maneuvers per patient was 2.1.^2^ A survey at the Poniente Hospital, Almeria, Spain, of 37 BPPV patients reported carrying out 1 maneuver in 65% of patients, 2 maneuvers in 27% of patients, and 3 maneuvers in 8% of patients.^17^ Most patients had unilateral BPPV. A study in the city of São Paulo concurred with our findings; in this paper, 89.1% of BPPV patients had single canal unilateral disease.^21^

Use of the DHI in the study sample revealed that dizziness negatively affected the QoL of patients in all dimensions of daily life. The physical scores were the most compromised aspect, followed by the functional and the emotional aspects. Castro^13^ suggested that the physical scores assess the relation between the onset/worsening of dizziness and eye, head and body movements of patients. The functional aspects investigate the effect of dizziness on specific eye, head and body movements, focusing in the subject's ability to carry out professional, household, social and leisure activities, and his or her independence in performing specific tasks such as walking independently and walking across the house in the dark. The emotional scores of the DHI investigate the possibility of dizziness having worsened the QoL of patients and giving rise to frustration, fear of leaving the house unaccompanied, fear of staying alone at home, concerns with the self-image, concentration disorders, feelings of incapacity, changes in family and social relationships, and depression.

The finding that pre-treatment physical scores were higher, followed by the functional and emotional scores, in BPPV patients is supported in the literature.^13^, ^14^, ^18^ Other authors^13^, ^14^, ^18^ have found that physical functions were more affected by vertigo than emotional and functional aspects.

The functional scores were second in the study sample with respect to the effects of dizziness on the QoL. These findings are similar to those of Castro (2007)^13^ who translated and adapted the DHI for the Brazilian population with chronic dizziness.

Emotional aspects were also affected in the study sample. These findings relate to psychological issues, such as how subjects feel about the opinions of others and issues such as depression. Paiva and Kuhn^22^ found that psychological symptoms were highly prevalent and associated with dizziness; anguish was present in 47.38% of patients, followed by anxiety (29.71%), fear (23.42%), and depression (12.58%). A study of patients with Ménière's disease showed that the mean emotional scores were 22.51 points during a crisis and 9.40 out of crises.^14^ A study on the progression of treatment for BPPV using Epley's maneuver showed that the emotional score was 6.72 before therapy and 4.94 after the treatment;^17^ these findings are similar to our results in which emotional scores decreased after treatment, from 13.2 to 3.2 points.

There was a statistically significant difference in the impact of dizziness on the QoL of patients before and after Epley's maneuver in all aspects (physical, functional and emotional). This demonstrates a positive effect of this maneuver on the QoL of patients. The effectiveness of repositioning maneuvers for the treatment of BPPV was demonstrated in a meta-analysis of available clinical trials;^23^ the remission rates reached 78%. It is important to note that remission may occur spontaneously in one third of patients after three weeks regardless of therapy. Furthermore, recurrences may occur - estimated at 15% a year.^23^ Although no clinical trials have compared directly the treatment choices, repositioning maneuvers are faster and more practical than vestibular rehabilitation therapies; there are also no significant adverse effects, especially when compared to drug therapy.

Studies on the factors affecting the QoL of patients with dizziness are relevant for clinical reasons; when placed alongside with the results of vestibular testing, professionals are able to better define the best approach by taking into account the changes in each patient with vertigo. The DHI may also be an interesting tool for checking the benefits and efficacy of conventional vestibular rehabilitation; it may be applied before and after therapy, which increases patient compliance - each subject may check his or her own difficulties in the questionnaire. Healthcare professionals in vestibular evaluation and rehabilitation should bear in mind that recognizing the negative effects on the QoL of patients with vertigo may be an important step in the rehabilitation process. This is a new supportive approach that has been used more often in medical practice.

## CONCLUSION

In out sample, Epley's maneuver resulted in a positive impact on the QoL on the physical, functional and emotional levels. The DHI score differences in BPPV patients before and after the repositioning maneuver were statistically significant.

Studies using the Brazilian DHI or other specific or non-specific questionnaires should be carried out to increase awareness of the self-perception about the consequences of dizziness in BPPV patients; the progression of patients undergoing several therapeutic approaches should also be investigated.
